# Nutrition, the digestive system and immunity in COVID-19 infection 

**Published:** 2020

**Authors:** Justine Bold, Miranda Harris, Lindsey Fellows, Manal Chouchane

**Affiliations:** 1 *School of Allied Health & Community, University of Worcester, Worcester, WR2 6AJ, UK*; 2 *Centre for Medical Education, Medical School, Cardiff University, Cardiff, CF14 4YS, UK *

**Keywords:** Obesity, COVID-19, Nutritional status, Vitamin D, Microbiome, Multidisciplinary, Supplementation, Practice

## Abstract

The current review aimed to synthesize the literature on the complex relationship between food consumption and nutritional status as well as the digestive system in order to examine the relationship between immunity and potential responses to COVID-19 infection. The goal is to help inform the many healthcare professionals working with COVID-19 patients. A literature search was performed on PubMed, Scopus, and EMBASE databases. Hand searches were also undertaken using Google and reference lists to identify recent evidence. Studies were critically appraised, and the findings were analyzed by narrative synthesis. Nutritional status can impact immunity in several ways, including affecting susceptibility to infection, severity of disease, and recovery time, and is therefore a significant consideration in the management of COVID-19. COVID-19 can also impact digestive function, which can further impact nutritional status. The role of Vitamin D deficiency in vulnerability to severe respiratory infections, including COVID-19, has been recognized, and it may have a role in treatment where deficiency is indicated. Healthcare professionals should be aware that obesity may be accompanied by micronutrient malnutrition including vitamin D deficiency and alterations in the microbiome and inflammatory responses, which can further impact immunity and disease severity. Multidisciplinary team-work is recommended in the management of patients with COVID-19, and approaches should include a consideration of nutritional status (both macronutrients and micronutrients), body weight, and gastrointestinal signs and symptom.

## Introduction

 The COVID-19 pandemic is a novel infection and a major global healthcare challenge which highlights the need for cost-effective strategies that both prevent complications and support recovery, reducing recovery times and complications wherever possible. The current review aims to consider the relationship between COVID-19 infection and nutritional status, including obesity, and the broader impact it may have on both digestive function and gastrointestinal (GI) symptoms of COVID-19 infection. It is hoped this will inform all areas of healthcare practice and increase awareness of the importance of considering nutrition in a multidisciplinary approach. 

**Method and Approach**

A narrative approach has been used to synthesize findings from a literature search using search terms such as COVID-19 and nutritional status, obesity, malnutrition, nutritional deficiency, vitamin D, and microbiome. PubMed, Scopus and EMBASE databases were used and hand searches were also undertaken using Google and reference lists to identify recent evidence. Studies were critically appraised, and the findings were analyzed by narrative synthesis.

**Malnutrition**

The nutritional status of populations is recognized as a key factor influencing resilience against destabilization in the current COVID-19 pandemic (1). Community resilience is generally considered to be the ability to recover from adverse events (2). Malnutrition is an established risk factor for lowered immunity (3), and a hospital stay can increase a patient’s chances of both complications and re-admission after discharge (4). Malnutrition can occur in both energy and macronutrient terms (carbohydrates, proteins, fats) and may also occur as micronutrient (vitamins and minerals) deficiency, which can potentially co-exist with obesity, where someone is potentially overfed in terms of energy (Kcal) but deficient in one or more micronutrients. A study published in 2017 reported on data from 1990-2017 and concluded that dietary factors were linked to 11 million deaths and 255 million disability-adjusted life-years across the world in 2017 (5).

Any infection outcome is affected by nutritional status, due to effects on both innate and adaptive immune responses (6). Vitamins B, C, and E alongside iron, selenium, and zinc play a supporting role in immunocompetence (7, 8), and any chronic shortages in these micronutrients may further impair immune function through cell activation and changes in signaling, molecule production, and gene expression (9). It should also be remembered that diet (and particularly components such as fiber) influences gut microbial composition which helps to promote immune responses in the body (10). 

**Vitamin D Status**

Vitamin D may protect against the risk of contracting influenza (11), and vitamin D deficiency is associated with a higher risk of community-acquired pneumonia (12) and GI disorders such as inflammatory bowel disease (13). It also seems to be an important factor in other viral respiratory infections. Alipio (14) presented significant data (*p*<0.001) regarding vitamin D status and the severity of SARS symptoms observed in patients admitted to three hospitals in South Asia. The data indicated that the majority of patients with mild symptoms (n=85.5%) presented with serum OH(D) of 31.2 ng/mmol (normal vitamin D status), with only 3.6% of patients in this category presenting with severe and critical symptoms (14). 

Vitamin D deficiency is common in people who are housebound and not going outside as vitamin D is synthesized through skin exposure to sunlight. Thus, a population in quarantine or lockdown may experience lowered levels of vitamin D. Moreover, skin pigmentation also affects production levels; skin with more pigmentation will produce less, so deficiency is more common in people with darker skin (15). Debates about whether this is implicated in higher death rates from COVID-19 infection among Black, Asian, and minority ethnic people (16) are gaining momentum. Both obesity and Type 2 diabetes (T2D) are also associated with high rates of vitamin D deficiency (17). It is also known that people with diabetes and obesity are more vulnerable to severe disease with COVID-19 infection. It seems vitamin D deficiency is relatively commonplace, as over a billion people globally are thought to be affected (18). In the UK, a country with high COVID-19 death rates, around a third of adults appear to be deficient in vitamin D. The National Diet and Nutrition Survey analysis of the last 9 years reported that in the UK from January to March, 19% of children aged 4 to 10 years, 37% of children aged 11 to 18 years, and 29% of adults had low Vitamin D levels (25-OHD below 25nmol/L), indicating a risk of 

**Stress**

Physical trauma is known to cause injury and dysfunction of the gut as well as increased intestinal permeability (20). Psychological stress also impacts gut dysbiosis and permeability by increasing the corticotropin-releasing hormone and its effect on TNFα (21), as well as other pro-inflammatory cytokines, which is evident in viral infections. The effect of being critically ill may compound an already compromised GI tract, and it is known that gut dysfunction can contribute to multi-organ dysfunction syndrome (20). Other GI mechanisms relevant to the pathophysiology of viral infection include the reduction of butyrate, which helps to maintain the gut barrier and is known for its anti-inflammatory effects, and the elevation of circulating lipopolysaccharides from the increase in gut permeability (22). This leads to the upregulation of inflammatory cytokines, which triggers systemic inflammation (23, 24). The consequential impact on mitochondrial function together with immune system responses further demonstrate the potential effects of COVID-19 on a range of interconnected body systems (25).

**Microbiome and Immunity**

Gut microbiota influences the maturation and development of immune cells such as dendritic cells and T cells, while also regulating the synthesis of the cytokines IL-10 (suppresses Th-1 dominant pro-inflammatory response) and TGF-ß (promotes the development of regulatory T cells) (26). Gut microbiota can also have indirect modulatory effects on inflammation including the production of short chain fatty acids (SCFA) (27) and the promotion of the synthesis of antimicrobial peptides (28). Thus, the interaction between the host’s microbiome and the immune system is complex due to the many pathways by which the microbiota can modulate immune responses to pathogens. 

*Lactobacillus* species specifically have been recognized for their role in stimulating the innate immune response by acting as ligands for Toll-like receptors (TLR) and activating important signaling pathways such as NF-kß, mitogen-activated protein kinase, and peroxisome proliferator-activated receptor gamma, all of which are involved in regulating cellular behaviors such as inflammatory responses (29). Furthermore, other species have been found to produce substances which can cross epithelial cell membranes and bind to intracellular receptors known as nucleotide-binding oligomerization domain receptors (NLRs) (30). NLR activation is responsible for regulating inflammatory responses by acting on the NF-kß and peroxisome proliferator-activated receptor gamma signaling pathways. TLRs and NLRs are associated with activating caspase 1, an enzyme involved in the maturation of the pro-inflammatory cytokines IL-1ß and IL-18 (31). Such pro-inflammatory effects have been suggested to serve physiological benefits such as immune system response ‘priming’, which can promote the host’s defense against pathogens (29).

Decreased microbiome biodiversity resulting from the lack of exposure to microorganisms due to excessive cleanliness (the ‘Hygiene Hypothesis’) (32), increased antibiotic use, and poor diet have been suggested to be major contributing factors in the increasing levels of inflammatory disorders. An imbalance in gut flora can trigger an inflammatory immune response, causing intestinal hyperpermeability, which in turn can trigger a further localized inflammatory response within the gut (33), creating a vicious cycle of inflammatory responses. SARS-Cov-2 causes lung infections by binding to angiotensin-converting enzyme 2 (ACE2) receptors within alveolar epithelial cells which, interestingly, are also expressed within intestinal enterocytes. Furthermore, SARS-Cov2 RNA was reported to be found in the feces of infected individuals (34). These factors along with the fact that microbiome diversity is reduced in older age, and it is the elderly patients who have suffered fatally from COVID-19, clearly indicates a possible link between COVID-19 immune response and gut microbiome.

**COVID-19 GI Symptoms**

Evidence suggests GI symptoms including nausea, loss of appetite, diarrhea and vomiting, and abdominal pain have been adduced in approximately a third of COVID-19 patients (35-38). More recently, olfactory and gustatory dysfunction have been reported as common symptoms, which may affect appetite (90).

**How the virus enters the cell**

Entry of the coronavirus into the cytoplasm of the host cell is initiated by the attachment of the receptor binding domain of the viral glycoprotein known as the spike (S) protein to the membrane protein ACE2. Transmembrane serine protease (TMPRSS) 2 facilitates entry into the cell by cleaving the S protein. As previously mentioned, ACE2 expression has been detected on the enterocytes of the small intestine, more so on the crypt epithelium and less so on the surface epithelium of the colon (34). TMPRSS2 has been observed on surface and crypt epithelia in the colon but is less obvious in the ileum and only on the crypt epithelium (39).

**Obesity**

Obesity, classified as a body mass index (BMI) > 30kg/m^2^, is recognized as one of the most significant public health problems in the world with approximately two billion overweight men and women (39% of adults aged 18 and over), of which 650 million are obese (40). If current trends of prevalence continue, 2.7 billion adults will be overweight by 2025, and one billion of these will be obese (40). One possible explanation of the virulence factor for COVID-19 in obesity and adverse outcomes is the rising issue of micronutrient and protein deficiency resulting from a monotonous diet often seen in a medical condition known as sarcopenic obesity, where low muscle mass and obesity coexist (41). Not only is there an increase in low-grade systemic inflammation due to excess adipose tissue, but the direct effect of vitamin deficiency such as A and D can lead to compromised functioning of antibody-secreting cells (42) and T-cell proliferation (43), respectively.

**Obesity as a Risk Factor for Severe Disease in COVID-19**

Obesity has been identified by the World Health Organization as a serious risk factor for COVID-19 outcomes and for poor adverse outcomes for COVID-19 (44). Increasing risks have been associated with increasing BMI (45). In a New York hospital cohort study (n=3615), obesity was cited as a risk factor for treatment escalation (46) and for admission to intensive care units (ICU) in the United Kingdom (UK). A study by the UK Intensive Care National Audit and Research Centre reported that over 73% of those in ICUs were overweight or obese (34.6% and 38.5%, respectively) (47). Obesity was recognized as a risk factor for in-hospital mortality after adjusting for other comorbidities in an observational, prospective cohort study in the UK (n=16,749) (48). 

Although obesity is associated with severe forms of COVID-19, the prevalence of severe obesity (BMI >35kg/m^2^) reported in ICUs may reflect the local prevalence of obesity as seen in two seminal studies in France, one in Lille, where the local prevalence was reported as 28.2% (45), and one in Lyon, where the prevalence was 11.3%. The requirement for invasive mechanical ventilation (IMV) compared to lean subjects reflected these differences with 68.6% and 58.4%, respectively. In a retrospective cohort study, obesity prevalence was high among ICU patients (44), and in other research, patients with a BMI between 30-34.9kg/m^2^ were 1.8 times more likely than those with a BMI <30kg/m^2^ to be admitted to the ICU (46). This is more of an issue in the USA and UK (countries which have high mortality rates in the current pandemic) because of the high prevalence of obesity (42% and 29%, respectively) (49, 50)

Obesity is a risk factor for impaired metabolic function, such as insulin resistance and T2D (51). The metabolic effect of excessive fat may result in reduced pancreatic beta cell performance, which may be compounded by the direct effect of COVID-19 on function (52). Inflammation of adipose tissue results in other metabolic dysfunctions such as dyslipidemia, hypertension, and cardiovascular disease (CVD) (51, 53). Although comorbidities for obesity such as heart and lung disease, hypertension, and T2D may confound results, severe obesity has been associated with ICU admissions and obesity associated with the use of IMV (both reaching statistical significance) (54).

**Obesity and Immune Function**

Obesity may predispose adults to impaired immune function and increased susceptibility to infection (51). Multiple factors are implicated, such as chronic inflammatory status, delayed immune response, and the complex relationships and interactions between adipose tissue and the immune system (55). High levels of ACE2-expressing cells are found in adipose tissue, and because obese individuals have more adipose tissue, this could elucidate that they have a greater amount of ACE2. COVID-19 has an enhanced affinity for ACE2 (53), which is a supposed receptor for the entry of COVID-19 into host cells (56). 

Adipose tissue may contribute to the progression of COVID-19 by other mechanisms, including the imbalance between anti-and pro-inflammatory cytokines, particularly adipokines; overexpression of the latter may lead to aberrant chemotaxis and abnormal macrophage differentiation, further compromising immune function (55). Other innate responses associated with obesity lead to the excessive release of pro-inflammatory cytokines such as IL-6 (55), TNFα, IFNý, and IL-2 and higher levels of circulating C-reactive protein (53). Elevated IL-6 levels have been correlated with ICU admission, respiratory failure, and adverse outcomes (39).

**Obesity and the Microbiota**

Studies have shown that obesity is associated with changes in the assortment of microorganisms inhabiting the GI tract, which include viral, fungal, bacterial, and single-celled microorganisms called arcaea, collectively known as the gut microbiota, and that obese adults have less diversity and richness in the bacterial composition (57-59). As previously mentioned, gut bacteria play a vital role in protection against pathogenic microorganisms, immune modulation, and other functions such as digestion and metabolism and SCFA production (60-62).

*Firmicutes and Bacteroidetes* make up approximately 90% of the total microorganisms with some research reporting an increased ratio of *Firmicutes* to *Bacteroidetes* in the feces of obese subjects (57, 63, 64); however, a systematic review was equivocal in its overview of the association between BMI and microbiota (65) with some studies supporting this theory and others finding no association, although this may be explained by differing methodological approaches. 

Recent evidence suggests the presence of microorganisms such as* Bacteroidetes,* *Firmicutes,* and *Proteobacteria* in the lungs and that a bidirectional relationship exists between the lungs and the gut (66). Dysbiosis, which results from a lack of gut diversity, has been associated with many diseases, including acute respiratory distress syndrome and sepsis (67).

2.4. Obesity and Dietary Habits

Obesity is affected by unhealthy eating habits, as seen in a cross-sectional study (n=1557) that looked at the association of abdominal obesity with food inadequacy and physical activity (68). They reported a lower likelihood of abdominal adiposity in those who consumed a minimum of three portions of fruit a day and did not exceed the upper limit of saturated fat (35% and 28%, respectively). Furthermore, in data from a longitudinal study n=50, improvements were found in weight, body fat, and waist circumference on a diet of fruits, vegetables, and grains containing 30g fiber/day compared to controls (69). Normal weight adults (n=52) consumed 43% more complex carbohydrates and 33% more fiber than overweight/obese adults when matched for gender, age, and physical activity (77). 

Diets that include high fat and low fiber can impact the diversity of the human gut microbiota, even over a few days (70). Conversely, a fiber-rich diet may have a positive effect on the gut microbiota and improve both metabolic and immune markers; for example, non-digestible carbohydrates such as whole grains may reduce pro-inflammatory cytokine IL-6 and insulin resistance (71). Interestingly, switching from a Western style diet of high fat/high sugar refined carbohydrates to a low-fat diet rich in plant-polysaccharides may influence the composition of microbiota, once again within a relatively short time, this time to the benefit of the microbiota (42). In addition, probiotics found in fermented foods may further reduce inflammation and help to regulate innate immunity (72). Anti-inflammatory marker IL-10 may be increased through the intake of prebiotics, increasing the production of SCFAs and improving the health of the GI-associated lymphoid tissue. Not only gut microbiota, but also lung microbiota is improved, thus offering a potential strategy for improving clinical outcomes of COVID-19 (41). 

**Melatonin**

Melatonin, released by the pineal gland at nighttime, has long been associated with the adjustment of the circadian phase to the environment, known as circadian rhythm entrainment; however, it also has antioxidant and anti-inflammatory properties, the latter due to its close relationship with mitochondrial function (73). The melatonergic pathway, which starts with the conversion of tryptophan to serotonin, then to N-acetylserotonin, and finally to melatonin, has created some interest due to its interaction with COVID-19 and other viruses (25). Elevated levels of stress may affect the availability of tryptophan for the melatonergic pathway, thus causing circadian (74, 75) and mitochondrial dysregulation (76). Pre-existing medical conditions mentioned previously have been linked to both of these regulatory mechanisms as well as to gut dysbiosis and increased gut permeability.

**Supportive Nutritional Interventions**

*Multidisciplinary Team Practice*

This review has indicated clear links between nutrition status (specifically micronutrient insufficiency and deficiency), compromised gut function, and the effects of obesity on viral severity, including SARS, CAP, and now COVID-19. Hence, a multidisciplinary approach is likely to be of benefit in terms of patient outcomes. 

Health services should adapt to prioritize reducing obesity and weight loss in general, given the inverse correlation between obesity/excess weight and COVID-19 severity. In response to this, the UK government launched an obesity strategy, labeled the COVID-19 ‘wake-up-call’, in July 2020 (81). The preventative strategies outlined include general practitioners being incentivized to prescribe activity and exercise to enhance physical fitness and primary care healthcare staff being trained as ‘healthy weight coaches’ to provide more specialist support to those in need (81). 

Allied health professionals with nutrition expertise such as dieticians, nutritionists, and nutritional therapists may also have a role in supporting individuals in their recovery from COVID-19 or in advising those seeking preventative or ‘prehabilitation’ healthy eating advice or supplement regimes (85). They can also help support individuals with weight loss. Ostensibly, these nutrition professionals will be required to assess the nutritional status and check drug-nutrient interactions of individuals in the first instance, before they provide safe guidance on nutritional supplementation. Since the start of the pandemic, claims, often unfounded and unsafe, of how specific foods and herbs may provide protection from COVID-19 have been circulating via social media (1). Thus, nutrition professionals have the added responsibility of dispelling any myths spread during the pandemic and ensuring safe intake of immune supporting nutrients (1). 

*Nutritional supplementation as Adjunct Treatment in COVID-19*

In supporting patients with COVID -19, vitamin D has the potential to be used as an adjunct treatment to medication. A variety of doses have been used to treat SARS and CAP to date, with therapeutic applications varying from 30-2500 μg/d (77, 78). Indeed, the results of a study currently ongoing at University Hospital, Angers (France), with COVID-19 patients ≥70 years of age are awaited. The hypothesis seeks to prove that high dose (2 x 2000,000 IU/d or 2 x 5 mg/d) vitamin D is more effective on improved prognosis when compared to a standard dose (50,000IU/d or 125 μg/d).

Evidence also exists in support of the probiotic *Lactobacillus Rhamanous,* which can be given as an oral supplement, and its efficacy in helping patients avoid being ventilated as well as in treating viral respiratory infection. *Bifidobacterium* appears to improve immune function and intestinal microbiota (79, 80). Both strains support further consideration for adjunct treatments in addressing possible dysbiosis and improving microbiome diversity.Vitamin C is known to be immunomodulatory. Moreover, antimicrobial and high-dose intravenous vitamin C is being researched for its potential in blocking the cytokine storm associated with COVID-19 infection (91). 

*Preventative Nutritional Intake and Supplementation for the General Population*

The debate regarding standardized, population-wide supplementation is ongoing, and the COVID-19 pandemic has certainly raised more questions around this topic. For example, the current UK recommendations for vitamin D supplementation of 10 μg/d are based on the role of vitamin D in bone health and calcium metabolism and do not take into account its role in immune regulation (82). Furthermore, this recommendation is for everyone aged 1 and older (8.5 μg/d birth to 1 year), with supplementation recommended in the winter months when sunlight is reduced. While not discounting the potential risks associated with higher intakes of vitamin D (83), this review has presented evidence linking the impact of modern day life, e.g., poor eating habits, stress, lack of microbiome diversity, and being overweight or obese, to nutritional insufficiency. 

Indeed, it is apparent that levels of micronutrient intake above those set by public health bodies (such as the RDA in the USA or the RNI in the UK) are required to support optimal function of the immune system, with recommendations for supplementation of vitamin D to be above the RDA (84). Similarly, this implication can be made to vitamin C, with the current UK RNI for adults being 40 mg/day (set at a level to prevent deficiency in the majority 97.5% of the population) (86). Vitamin C deficiency is linked to severe respiratory tract infections and overall disease severity, and plasma levels are known to be affected by infection (84). The UK RNI is significantly less than guidance in other countries, and new evidence is emerging to optimally support immune function (84, 87). Table 1 outlines a summary of the recommendations for supplementation of vitamins D and C based on current evidence.

**Table 1:** Recommended intakes of vitamins D and C to support optimal immunity for the wider population

Nutrient	Justification	Recommendation
Vitamin D	Revision of the current UK guidelines is advocated (82). 10 μg/d is not sufficient for individuals in at-risk groups at any time (82). 10 μg/d is not sufficient for individuals between October-April in cold, Northern Hemisphere countries (77).	Daily intake of 50 μg/d (84, 85) [Daily intake of 50-125 μg/d has been advised dependent on age, risk, and time of year (11).]
Vitamin C	40 mg/d is not sufficient for UK adults (86). EU guidance for adults is 95 mg/d (87). Higher daily doses (≥200mg/d) are required for optimal immune function (84).	Daily intake of 200 mg/d (84) [Daily intake of 1-2 g/d for individuals who are unwell (84)]

## Conflict of interests

The authors declare that they have no conflict of interest.
